# 12-month clinical outcomes of combined phacoemulsification and ab interno trabeculectomy for open-angle glaucoma in the United Kingdom

**DOI:** 10.1371/journal.pone.0252826

**Published:** 2021-06-17

**Authors:** Ejaz Ansari, Deva Loganathan

**Affiliations:** 1 Maidstone & Tunbridge Wells NHS Trust and University of Kent, Canterbury, United Kingdom; 2 Maidstone & Tunbridge Wells NHS Trust, Maidstone, United Kingdom; Cairo University Kasr Alainy Faculty of Medicine, EGYPT

## Abstract

**Background/Objectives:**

To describe intraocular pressure (IOP) and ocular hypotensive medication outcomes of combined phacoemulsification and ab interno trabeculectomy with the Kahook Dual Blade (KDB; New World Medical, Inc, Rancho Cucamonga, CA) in adults with cataract and open-angle glaucoma (OAG).

**Subjects/Methods:**

Retrospective chart review of existing medical records. Data collected included intraocular pressure (IOP) and IOP-lowering medication use preoperatively and through up to 24 months postoperatively. Paired t-tests were utilized to compare preoperative to postoperative mean IOP and mean medications used.

**Results:**

Data from 32 eyes of 26 subjects were analyzed. Subjects were predominantly Caucasian (25/26) had mean (standard error) age of 79.3 (1.2) years, and eyes had moderate-advanced OAG (mean visual field mean deviation -8.3 [1.3] dB). Mean IOP was 19.8 (0.8) mmHg at baseline and 15.5 (0.6) mmHg (p<0.0001) after mean follow-up of 11.5 (1.0) months; IOP reductions of ≥20% were achieved in 20/32 eyes (62.5%). Mean medication use declined from 2.4 (0.2) medications per eye at baseline to 0.5 (0.2) at last follow-up (p<0.0001); 23/32 eyes (71.9%) were medication-free at last follow-up. No vision-threatening complications were observed.

**Conclusions:**

Combined phacoemulsification and ab interno trabeculectomy with the KDB safely provided mean IOP reductions of 21.7% and mean IOP medication reductions of 83% after mean follow-up of 12 months in eyes with moderate to advanced OAG. This procedure provides medication-independence in most eyes with statistically and clinically significant IOP reductions.

## Introduction

Glaucoma is the leading cause of irreversible blindness worldwide [1], and the prevalence of primary open-angle glaucoma (POAG) among adults over age 65 years in England and Wales—the most common form of glaucoma in the region [2]—is 3% [3] and in Northern Ireland is 2.83% [4]. Vision loss or blindness from glaucoma represents approximately 7–15% of all vision loss/blindness in the United Kingdom (UK) [5–7], resulting in direct health care costs of approximately £200 million annually [5].

Primary therapeutic options for POAG include both medical and laser therapy options, both of which effectively lower intraocular pressure (IOP) and prevent glaucoma-related vision loss [8]. Historically, surgical therapies such as ab externo trabeculectomy or tube-shunt implantation have been reserved for more severe or recalcitrant cases. More recently, the development of numerous minimally invasive surgical options that lower IOP somewhat less than older procedures but with more favorable safety profiles has expanded indications for surgery earlier in the disease course and in the treatment cascade [9–11]. Many of these procedures can be performed either as standalone surgery in eyes with inadequate IOP control or in combination with cataract surgery to reduce IOP and/or the medication burden in eyes with coincident glaucoma.

One such procedure is an ab interno trabeculectomy. In this procedure, a strip of trabecular meshwork (TM) is removed from the anterior chamber angle of the eye using a specialized instrument—the Kahook Dual Blade (KDB, New World Medical, Rancho Cucamonga, CA)—to facilitate aqueous humor drainage into Schlemm’s canal [12]. Ab interno trabeculectomy can be performed as a standalone procedure or in combination with phacoemulsification cataract surgery (KDB-phaco). In previous studies conducted primary in the United States, KDB-phaco produced IOP reductions ranging from 12–27% and medication reductions of 21–71% [13–22].

In this paper, we present clinical outcomes of KDB-phaco through up to 24 months of follow-up in an academic practice in the United Kingdom.

## Subjects and methods

This was a retrospective analysis of existing data drawn from the medical records of qualifying patients. The protocol for data collection and analysis was reviewed and approved by the Maidstone and Tunbridge Wells NHS Trust Research and Development Committee, which granted a waiver of consent.

Subjects included in this analysis were adults aged 18 years or older with visually significant cataract and medically-controlled open-angle glaucoma who underwent KDB-phaco in one or both eyes at the Maidstone and Tunbridge Wells Hospitals, England, between September 2017 and May 2019. Patients with glaucomas other than open-angle glaucoma, and those with corneal pathology precluding accurate assessment of IOP by applanation tonometry, were excluded. Prior incisional glaucoma surgery (prior iridotomy was acceptable if angles were open), presence of vitreous in the anterior chamber, presence of intraocular silicone oil, clinically significant inflammation or infection in the study eye within 30 days prior to the preoperative visit, active ophthalmic disease/disorder that could confound study results and impaired episcleral venous drainage were also excluded. All qualifying patients within this time frame were included to preclude selection bias.

The procedure has been described previously [12, 13]. Briefly, under topical tetracaine 1% and intracameral lidocaine 1% anesthesia and following standard phacoemulsification and intraocular lens implantation (EyeCee One, Bausch & Lomb UK, Kingston-Upon-Thames, England), the KDB was introduced into the anterior chamber through the temporal clear corneal surgical incision and advanced to the nasal angle. The TM was engaged with the instrument’s tip, which was advanced until the instrument’s heel was seated within Schlemm’s canal. The instrument was then advanced along Schlemm’s canal for the intended extent of TM excision; during advancement, TM tissue was stretched and elevated up the integrated ramp to two parallel blades that excised a strip of TM, which was removed with forceps or with the KDB itself. At the completion of surgery, all ocular hypotensive therapy was discontinued, and dexamethasone 0.1%, nepafenac 0.1%, and pilocarpine 2% each dosed x4 daily for 4 weeks were prescribed. Data retrieved from electronic health records included demographic and baseline glaucoma status data, visual acuity and IOP and medication data at baseline and every postoperative visit, and adverse events occurring intra- or postoperatively. Data were drawn from the preoperative assessment visit, the surgical visit, and the follow-up visits closest in time to 2 weeks, 6 weeks, 3–6 months, and the last follow-up visit for patients followed beyond 4 months and up to 24 months.

The primary outcomes were reductions from baseline in IOP and IOP-lowering medications at each postoperative time point. These were assessed using paired t-tests, with p = 0.05 taken as the level of significance. Visual acuity (VA) changes from baseline were similarly assessed using the logMAR form of VA. Safety assessment included the nature, frequency, and timing of adverse events. As no pre-specified hypotheses were being tested, *a priori* power and sample size calculations were not undertaken, and the sample size was set arbitrarily by the number of cases available for analysis at the time of data acquisition.

## Results

This analysis includes data from 32 eyes of 26 subjects undergoing combined KDB-phacoemulsification. Demographic and baseline glaucoma status data are given in Table 1. Patients were Caucasian (96.2%, 25/26), were on average 79.3 (1.4) years of age, and had moderate or advanced OAG (mean visual field mean deviation -8.3 [1.3] dB). Mean follow-up was 11.5 (1.0) months (range 6–24 months).

**Table 1:** Demographics and baseline glaucoma status in 32 eyes of 26 subjects.

Parameter	Value
Subject-Level (n = 26)	
Age (yr), mean (SE)	79.3 (1.4)
Gender, n (%)	
Male	10 (38.4)
Female	16 (61.6)
Ethnicity, n (%)	
Caucasian	25 (96.1)
Asian	1 (3.9)
Eye-Level (n = 32)	
Operative eye, n (%)	
Right	16 (50)
Left	16 (50)
Visual field mean deviation (dB), mean (SE)	-8.3 (1.3)
Cup-disc ratio, mean (SE)	0.75 (0.0)
Follow-up, mean (SE) [range] (months)	11.9 (1.0) [4–24]

Mean IOP data at each time point is given in Table 2. Mean IOP was 19.8 (0.8) mmHg at baseline; significant IOP reductions were seen from Week 6 through the end of follow-up, ranging from 4.1–4.3 mmHg (p≤0.0002). At last follow-up, IOP reductions of ≥20% were achieved in 20/32 eyes (62.5%). Final IOP ≤18 mmHg was achieved in 84.4% of eyes (27/32) and IOP ≤ 15 mmHg was achieved in 56.3% of eyes (18/32) (Table 3).

**Table 2:** Intraocular pressure, medication, and visual acuity data at each time point.

	Baseline	Week 2	Week 6	Month 3–6	Last Follow-Up
Number of Eyes	32	12	19	32	32
Intraocular pressure					
Mean (SE), mmHg	19.8 (0.8)	17.9 (0.9)	15.1 (1.0)	15.6 (0.7)	15.5 (0.6)
Mean (SE) change from baseline, mmHg	---	-2.8 (1.5)	-4.0 (0.9)	-4.3 (0.9)	-4.3 (0.8)
Mean (SE) % change from baseline, %	---	-10.6 (5.8)	-20 (4.5)	-18.7 (4.2)	-18.9 (3.9)
p (mean change from baseline)	---	0.0945	0.0002	< .0001	< .0001
Medications					
Mean (SE), n	2.4 (0.2)	---	---	0.1 (0.0)	0.5 (0.2)
Mean (SE) change from baseline, n	---	---	---	-2.3 (0.2)	-1.9 (0.2)
Mean (SE) % change from baseline, %	---	---	---	-98.3 (1.2)	-82.5 (6.4)
P (mean change from baseline)	---	---	---	< .0001	< .0001
Best-corrected visual acuity					
Mean (SE), logMAR	0.52 (0.08)	0.25 (0.07)	0.11 (0.24)	0.11 (0.04)	0.09 (0.04)
Mean (SE) change from baseline, logMAR	---	-0.08 (0.08)	-0.47 (0.25)	-0.41 (0.09)	-0.42 (0.07)
Mean (SE) % change from baseline, %	---	-46.8 (24.4)	-74.1 (50.6)	-67.6 (12.3)	-84 (6.2)
P (mean change from baseline)	---	0.3504	0.0675	< .0001	< .0001

logMAR, logarithm of the minimum angle of resolution; mmHg, millimeters of Mercury; SE, standard error

**Table 3:** Pre-specified IOP and medication outcomes at last follow-up.

Number of eyes (n)	32
Proportion achieving IOP reduction ≥20% compared to baseline, n(%)	20 (62.5)
Proportion achieving IOP ≤18 mmHg, n (%)	27 (84.4)
Proportion achieving IOP ≤15 mmHg, n(%)	18 (56.3)
Proportion using ≥ 1 fewer medication compared to baseline, n(%)	28 (87.5)
Proportion medication-free, n (%)	23 (71.9)

IOP, intraocular pressure; mmHg, millimeters of Mercury

Mean medication use at each time point following stabilization of the medication regimen at Month 3 is also given in Table 2. The mean number of medications used per eye was 2.4 (0.2) at baseline, by Month 3–6 was reduced to 0.1 (0.0) (p<0.0001), and at last follow-up was 0.5 (0.2), an 82.5% reduction (p<0.0001). At last follow-up, the medication burden has been reduced by at least 1 medication in 87.5% of eyes (28/32), and 71.9% (23/32) were medication-free.

The procedure was well tolerated by all patients. Four eyes (12.5%) had transient hyphema either intraoperatively or within the first few postoperative days, all of which resolved spontaneously. No eyes required any additional glaucoma procedures during the observed follow-up period. Mean logMAR BCVA improved from 0.52 (0.8) to 0.09 (0.04) (p<0.0001); BCVA was improved (90.6% [29/32]) or unchanged (within 1 line; 9.4% [3/32]) in all eyes, with no eyes losing >1 lines of BCVA.

## Discussion

This study demonstrates that KDB-phaco, performed in eyes with both cataract and open-angle glaucoma, effectively lowers both IOP and the need for IOP-lowering medications in adult patients in the UK. The majority (72%) of these eyes—many with moderate or advanced glaucoma—were medication-free with IOP reductions of 20% or more (62.5%) at last follow-up (6–24 months postoperatively). The procedure was safe, with minimal and self-limited complications that were not sight threatening.

The IOP and medication reductions seen in this study (21.7% and 83%, respectively) are consistent with those reported in other populations. In studies of 6–12 months’ duration conducted largely in the US in eyes with predominantly OAG, KDB-phaco provided mean IOP reductions 12–27% and medication reductions of 21–71% [13–22]. Less is known about outcomes of KDB-phaco in international populations. A recently-reported retrospective study from Saudi Arabia of 10 standalone and 40 KDB-phaco cases found mean IOP reduction of 29% and mean medication reduction of 86% 4–7 months postoperatively [23]. In a mixed sample of subjects from the US, Mexico, and Switzerland, all with severe or refractory glaucoma, mean IOP reductions of 24% and medication reductions of 37% were reported at 6 months [24]. Overall, these international studies in patients of various ethnicities demonstrate consistency in the efficacy of the KDB-phaco procedure. In this study, reflective of the authors’ practice, effort was made to reduce medications whenever possible postoperatively while still maintaining target IOP, in an effort to improve patients’ quality of life and minimize ocular surface symptoms associated with topical ocular hypotensive therapy as a means of sparing the conjunctiva for possible future bleb-based surgery [25–27]. Had medications been discontinued less determinedly, IOP reductions may have been greater at the expense of medication reductions. Variations among investigators of prior studies in discontinuing medications postoperatively may explain in part the substantial inter-study variability in IOP and medication reductions reported.

Glaucoma and cataract both increase in prevalence with increasing age and frequently coexist. Elective cataract surgery affords an opportunity for the surgical redress of glaucoma, however until only recently the surgical options for glaucoma at the time of cataract surgery have been limited to full-thickness procedures—trabeculectomy and tube-shunt implantation—with safety profiles that limited their value as add-on procedures to cataract surgery in patients with moderate IOP or medication reduction goals. In recent years, a growing family of less invasive procedures has been developed for patients in whom modest IOP reductions, or reductions in the medication burden, are desirable [9–11]. These relatively safer procedures are generally less traumatic to ocular tissues and offer faster visual rehabilitation compared to trabeculectomy or tube-shunt implantation [28] and can thus be paired with cataract surgery for co-management of cataract and glaucoma. These procedures are generally classified into those that shunt aqueous humor into Schlemm’s canal, the suprachoroidal space, or the subconjunctival space, and have been extensively reviewed elsewhere [9–11, 28–32]. As the current study and previous studies discussed above demonstrate, the KDB-phaco procedure effectively reduces both IOP and the IOP-lowering medication burden at the time of cataract surgery while providing excellent visual rehabilitation. Significantly, the KDB-phaco procedure does not induce unexpected postoperative refractive errors [33].

This study’s key strength is its inclusion of a sample representing a population in which results of KDB-phaco have not been previously reported, broadening the evidence base for this procedure’s international value in addressing coexisting cataract and glaucoma. The length of follow-up—through up to 24 months—is also a strength given the chronicity of glaucoma and the relatively shorter duration (6–12 months) of most prior studies of ab interno trabeculectomy [13–22]. Its retrospective design is a limitation, although the risk of selection bias was minimized by including all consecutive eyes undergoing the procedure during the included time frame. This is also an uncontrolled study, and cataract surgery alone can lower both IOP and the medication burden, although the reductions expected from phacoemulsification alone—14% IOP reduction and 0.5 medication reduction at 1 year [34]—are not sufficient to account for the efficacy outcomes observed in our series.

In summary, the combination of phacoemulsification and ab interno trabeculectomy with the Kahook Dual Blade significantly lowered both IOP and the need for IOP-lowering medications, with excellent visual acuity rehabilitation, in patients with cataract and glaucoma in the United Kingdom.

## References

[1] Flaxman SR, Bourne RRA, Resnikoff S, et al. Global Causes of Blindness and Distance Vision Impairment 1990–2020: A Systematic Review and Meta-Analysis. The Lancet Global Health 2017. doi: 10.1016/S2214-109X(17)30393-5 29032195

[2] Chan MPY, Broadway DC, Khawaja AP, et al. Glaucoma and Intraocular Pressure in Epic-Norfolk Eye Study: Cross Sectional Study. BMJ 2017;358:j3889. doi: 10.1136/bmj.j3889 28903935 PMC5596699

[3] Minassian DC, Reidy A, Coffey M, Minassian A. Utility of Predictive Equations for Estimating the Prevalence and Incidence of Primary Open Angle Glaucoma in the Uk. Br J Ophthalmol 2000;84:1159–61. doi: 10.1136/bjo.84.10.1159 11004103 PMC1723266

[4] McCann P, Hogg R, Wright DM, et al. Glaucoma in the Northern Ireland Cohort for the Longitudinal Study of Ageing (Nicola): Cohort Profile, Prevalence, Awareness and Associations. Br J Ophthalmol 2020. 10.1136/bjophthalmol-2019-315330 32034006

[5] Pezzullo L, Streatfeild J, Simkiss P, Shickle D. The Economic Impact of Sight Loss and Blindness in the Uk Adult Population. BMC Health Serv Res 2018;18:63. doi: 10.1186/s12913-018-2836-0 29382329 PMC5791217

[6] Quartilho A, Simkiss P, Zekite A, Xing W, Wormald R, Bunce C. Leading Causes of Certifiable Visual Loss in England and Wales During the Year Ending 31 March 2013. Eye (Lond) 2016;30:602–7. doi: 10.1038/eye.2015.288 26821759 PMC5108547

[7] Bamashmus MA, Matlhaga B, Dutton GN. Causes of Blindness and Visual Impairment in the West of Scotland. Eye 2004;18:257–61. doi: 10.1038/sj.eye.6700606 15004574

[8] Gazzard G, Konstantakopoulou E, Garway-Heath D, et al. Selective Laser Trabeculoplasty Versus Eye Drops for First-Line Treatment of Ocular Hypertension and Glaucoma (Light): A Multicentre Randomised Controlled Trial. Lancet 2019;393:1505–16. doi: 10.1016/S0140-6736(18)32213-X 30862377 PMC6495367

[9] Richter GM, Coleman AL. Minimally Invasive Glaucoma Surgery: Current Status and Future Prospects. Clinical Ophthalmology 2016;10:189–206. doi: 10.2147/OPTH.S80490 26869753 PMC4734795

[10] Lavia C, Dallorto L, Maule M, Ceccarelli M, Fea AM. Minimally-Invasive Glaucoma Surgeries (MIGS) for Open Angle Glaucoma: A Systematic Review and Meta-Analysis. PLoS ONE 2017;12:e0183142. doi: 10.1371/journal.pone.0183142 28850575 PMC5574616

[11] Ansari E. An Update on Implants for Minimally Invasive Glaucoma Surgery (MIGS). Ophthalmology and therapy 2017;6:233–41. doi: 10.1007/s40123-017-0098-2 28730285 PMC5693836

[12] Seibold LK, Soohoo JR, Ammar DA, Kahook MY. Preclinical Investigation of Ab Interno Trabeculectomy Using a Novel Dual-Blade Device. Am J Ophthalmol 2013;155:524–9 e2. doi: 10.1016/j.ajo.2012.09.023 23218696

[13] Dorairaj SK, Seibold LK, Radcliffe NM, et al. 12-Month Outcomes of Goniotomy Performed Using the Kahook Dual Blade Combined with Cataract Surgery in Eyes with Medically Treated Glaucoma. Adv Ther 2018;35:1460–9. doi: 10.1007/s12325-018-0755-4 30078175 PMC6133141

[14] Dorairaj SK, Kahook MY, Williamson BK, Seibold LK, ElMallah MK, Singh IP. A Multicenter Retrospective Comparison of Goniotomy Versus Trabecular Bypass Device Implantation in Glaucoma Patients Undergoing Cataract Extraction. Clin Ophthalmol 2018;12:791–7. doi: 10.2147/OPTH.S158403 29750011 PMC5933398

[15] Greenwood MD, Seibold LK, Radcliffe NM, et al. Goniotomy with a Single-Use Dual Blade: Short-Term Results. J Cataract Refract Surg 2017;43:1197–201. doi: 10.1016/j.jcrs.2017.06.046 28991617

[16] Sieck EG, Epstein RS, Kennedy JB, et al. Outcomes of Kahook Dual Blade Goniotomy with and without Phacoemulsification Cataract Extraction. Ophthalmology Glaucoma 2018;1:75–81. doi: 10.1016/j.ogla.2018.06.006 32672636

[17] ElMallah MK, Seibold LK, Kahook MY, et al. 12-Month Retrospective Comparison of Kahook Dual Blade Excisional Goniotomy with Istent Trabecular Bypass Device Implantation in Glaucomatous Eyes at the Time of Cataract Surgery. Adv Ther 2019;36:2515–27. doi: 10.1007/s12325-019-01025-1 31317390 PMC6822852

[18] Hirabayashi MT, King JT, Lee D, An JA. Outcome of Phacoemulsification Combined with Excisional Goniotomy Using the Kahook Dual Blade in Severe Glaucoma Patients at 6 Months. Clin Ophthalmol 2019;13:715–21. doi: 10.2147/OPTH.S196105 31114149 PMC6488161

[19] Le C, Kazaryan S, Hubbell M, Zurakowski D, Ayyala RS. Surgical Outcomes of Phacoemulsification Followed by Istent Implantation Versus Goniotomy with the Kahook Dual Blade in Patients with Mild Primary Open-Angle Glaucoma with a Minimum of 12-Month Follow-Up. J Glaucoma 2019;28:411–4. doi: 10.1097/IJG.0000000000001143 31048639

[20] Kornmann HL, Fellman RL, Feuer WJ, et al. Early Results of Goniotomy with the Kahook Dual Blade, a Novel Device for the Treatment of Glaucoma. Clin Ophthalmol 2019;13:2369–76. doi: 10.2147/OPTH.S224643 31819362 PMC6896929

[21] Lee D, King J, Thomsen S, Hirabayashi M, An J. Comparison of Surgical Outcomes between Excisional Goniotomy Using the Kahook Dual Blade and Istent Trabecular Micro-Bypass Stent in Combination with Phacoemulsification. Clin Ophthalmol 2019;13:2097–102. doi: 10.2147/OPTH.S224109 31749607 PMC6818539

[22] Hirabayashi MT, Lee D, King JT, Thomsen S, An JA. Comparison of Surgical Outcomes of 360 Degrees Circumferential Trabeculotomy Versus Sectoral Excisional Goniotomy with the Kahook Dual Blade at 6 Months. Clin Ophthalmol 2019;13:2017–24. doi: 10.2147/OPTH.S208468 31686776 PMC6800543

[23] Barry M, Alahmadi MW, Alahmadi M, AlMuzaini A, AlMohammadi M. The Safety of the Kahook Dual Blade in the Surgical Treatment of Glaucoma. Cureus 2020;12:e6682. doi: 10.7759/cureus.6682 31976190 PMC6968824

[24] Salinas L, Chaudhary A, Berdahl JP, et al. Goniotomy Using the Kahook Dual Blade in Severe and Refractory Glaucoma: 6-Month Outcomes. J Glaucoma 2018;27:849–55. doi: 10.1097/IJG.0000000000001019 29979337

[25] Baudouin C, Labbe A, Liang H, Pauly A, Brignole-Baudouin F. Preservatives in Eyedrops: The Good, the Bad and the Ugly. Prog Retin Eye Res 2010. doi: 10.1016/j.preteyeres.2010.03.001 20302969

[26] Fechtner RD, Godfrey DG, Budenz D, Stewart JA, Stewart WC, Jasek MC. Prevalence of Ocular Surface Complaints in Patients with Glaucoma Using Topical Intraocular Pressure-Lowering Medications. Cornea 2010;29:618–21. doi: 10.1097/ICO.0b013e3181c325b2 20386433

[27] Leung EW, Medeiros FA, Weinreb RN. Prevalence of Ocular Surface Disease in Glaucoma Patients. J Glaucoma 2008;17:350–5. doi: 10.1097/IJG.0b013e31815c5f4f 18703943

[28] Saheb H, Ahmed II. Micro-Invasive Glaucoma Surgery: Current Perspectives and Future Directions. Curr Opin Ophthalmol 2012;23:96–104. doi: 10.1097/ICU.0b013e32834ff1e7 22249233

[29] Agrawal P, Bradshaw SE. Systematic Literature Review of Clinical and Economic Outcomes of Micro-Invasive Glaucoma Surgery (Migs) in Primary Open-Angle Glaucoma. Ophthalmology and Therapy 2018;7:49–73. doi: 10.1007/s40123-018-0131-0 29725860 PMC5997597

[30] Pillunat LE, Erb C, Junemann AG, Kimmich F. Micro-Invasive Glaucoma Surgery (Migs): A Review of Surgical Procedures Using Stents. Clin Ophthalmol 2017;11:1583–600. doi: 10.2147/OPTH.S135316 28919702 PMC5587165

[31] Shah M. Micro-Invasive Glaucoma Surgery—an Interventional Glaucoma Revolution. Eye and Vision 2019;6:29. doi: 10.1186/s40662-019-0154-1 31583261 PMC6766174

[32] Dick HB, Schultz T, Gerste RD. Miniaturization in Glaucoma Monitoring and Treatment: A Review of New Technologies That Require a Minimal Surgical Approach. Ophthalmology and therapy 2019;8:19–30. doi: 10.1007/s40123-019-0161-2 30725339 PMC6393261

[33] Sieck EG, Capitena Young CE, Epstein RS, et al. Refractive Outcomes among Glaucoma Patients Undergoing Phacoemulsification Cataract Extraction with and without Kahook Dual Blade Goniotomy. Eye and vision 2019;6:28. doi: 10.1186/s40662-019-0153-2 31548974 PMC6751845

[34] Armstrong JJ, Wasiuta T, Kiatos E, Malvankar-Mehta M, Hutnik CM. The Effects of Phacoemulsification on Intraocular Pressure and Topical Medication Use in Patients with Glaucoma: A Systematic Review and Meta-Analysis of 3-Year Data. J Glaucoma 2017;26:511–22. doi: 10.1097/IJG.0000000000000643 28333892

