# Supplementary material for: 12-month clinical outcomes of combined phacoemulsification and ab interno trabeculectomy for open-angle glaucoma in the United Kingdom
Source: PLoS One. 2021 Jun 17;16(6):e0252826. doi: 10.1371/journal.pone.0252826 (PMC8211240; doi:10.1371/journal.pone.0252826)
Supplement: Attachment — Submitted filename: PLOS1 respose to reviewers letter KDB.docx [file pone.0252826.s001.docx]

Editor-in-Chief

PLOS 1

14th May 2021

Dear Sir/ Madam

*Re: PONE-D-21-11179 PLOS 1 12-Month Clinical Outcomes of Combined Phacoemulsification and Ab Interno Trabeculectomy for Open-Angle Glaucoma in the United Kingdom*

Many thanks for the useful and constructive comments by the reviewers.

Response to Reviewers:

Response to Reviewer 1:

Variable follow-up of this cohort precludes any absolute descriptor of the follow-up period. We elected to include the mean follow-up in the title to provide readers with context. The mean is the standard summary statistic for continuously- and normally-distributed data. As for a phaco-only control group, we would be unable to collect such data as our practice is to perform phaco-MIGS in eyes such as these to reduce IOP and/or the medication burden. Thus, eyes undergoing phaco alone would be sufficiently different from study eyes as to introduce confounding factors. As for comparisons to eyes with more advanced glaucoma, we typically perform alternative procedures in those eyes.

Response to Reviewer 2:

1- the inclusion and exclusion criteria- these have been included in the text (see tracked)

2- a short description of the surgical technique, anaesthesia, IOL type*-* these have been included (see tracked).

Thank you in advance for your time and consideration.

Sincerely,

Ejaz Ansari, MD, for the research team
